# Structural Changes in High-Entropy Alloys CoCrFeNi and CoCrFeMnNi, Irradiated by He Ions at a Temperature of 700 °C

**DOI:** 10.3390/ma17174383

**Published:** 2024-09-05

**Authors:** Igor Ivanov, Bauyrzhan Amanzhulov, Vladimir Uglov, Sergey Zlotski, Alisher Kurakhmedov, Mikhail Koloberdin, Asset Sapar, Yerulan Ungarbayev, Maxim Zdorovets

**Affiliations:** 1 Institute of Nuclear Physics, Almaty 050032, Kazakhstan; igor.ivanov.inp@gmail.com (I.I.); koloberdin@inp.kz (M.K.);; 2 Engineering Profile Laboratory, L.N. Gumilyov Eurasian National University, Astana 010008, Kazakhstan; 3 Physical-Technical Faculty, L.N. Gumilyov Eurasian National University, Astana 010008, Kazakhstan; 4 Department of Solid State Physics, Belarusian State University, 220030 Minsk, Belarus; uglov@bsu.by (V.U.); zlotski@bsu.by (S.Z.)

**Keywords:** high-entropy alloys, radiation resistance, XRD analysis, microstress, blistering

## Abstract

High-entropy alloys (HEA) are promising structural materials that will successfully resist high-temperature irradiation with helium ions and radiation-induced swelling in new generations of nuclear reactors. In this paper, changes in the elemental and phase composition, surface morphology, and structure of CoCrFeNi and CoCrFeMnNi HEAs irradiated with He^2+^ ions at a temperature of 700 °C were studied. Structural studies were mainly conducted using the X-ray diffraction method. The formation of a porous surface structure with many microchannels (open blisters) was observed. The average diameter of the blisters in CoCrFeMnNi is around 1.3 times smaller than in CoCrFeNi. It was shown that HEAs’ elemental and phase compositions are stable under high-temperature irradiation. It was revealed that, in the region of the peak of implanted helium, high-temperature irradiation leads to the growth of tensile macrostresses in CoCrFeNi by 3.6 times and the formation of compressive macrostresses (−143 MPa) in CoCrFeMnNi; microstresses in the HEAs increase by 2.4 times; and the dislocation density value increases by 4.3 and 7.5 times for CoCrFeNi and CoCrFeMnNi, respectively. The formation of compressive macrostresses and a higher value of dislocation density indicate that the CoCrFeMnNi HEA tends to have greater radiation resistance compared to CoCrFeNi.

## 1. Introduction

The current challenges facing the scientific and technological part of nuclear energy are largely related to increasing the efficiency of nuclear power plants by increasing their operating temperatures. In the 4th generation of nuclear reactors, including fast reactors, the neutron load and temperatures will be higher than in the previous generation of reactors. In light water reactors, at temperatures of 250–400 °C and damage greater than 0.1 displacement per atom (dpa), the main damage is due to deterioration of fracture toughness, corrosion, radiation-induced creep, and in fast reactors, void swelling is added. The structural materials of the 4th generation reactors (such as fast reactors) must withstand the temperature of 300–1000 °C, damage doses for core structures of 30–200 dpa, helium concentrations up to 40 atoms per million, neutrons with energy of 1–3 MeV, and resist radiation swelling, embrittlement, and other effects that occur at elevated temperatures and damage doses [1,2].

High-entropy alloys (HEA) are alloys of five or more metals, with close to equiatomic concentrations of the main elements, where the concentration of each of them is at least 5–35 atomic percent (at.%), and the configurational entropy is ΔS_conf_ ≥ 1.5R (where R is universal gas constant), while for medium-entropy alloys (MEA) 1.0 ≤ ΔS_conf_ ≤ 1.5R [3]. HEAs based on FCC transition metals Ni, Co, Fe, Cr, and Mn, along with oxide dispersion-strengthened steels (ODS), are alternatives to such structural materials as austenitic and ferritic-martensitic steels, since they are less susceptible to void swelling and have enhanced recombination of point defects [4,5]. HEAs have high strength and hardness, and due to high configurational entropy and lattice distortions, combinations of component qualities, they can withstand high temperatures and large doses of radiation without significant deterioration in mechanical properties [5].

One of the important problems in reactor structural materials is the formation and deposition of gases insoluble in metals, such as hydrogen and helium. In thermal reactors, helium swelling is more pronounced than in fast reactors, and helium in reactors is formed due to transmutation reactions, such as neutron absorption by boron atoms or a two-step reaction of nickel-to-iron conversion [6,7]. Radiation-induced swelling is especially pronounced in the temperature range of 0.3–0.6 *T_m_*, where *T_m_* is the melting point of the alloy [5]. Helium released during fission reactions accumulates in metal systems due to its low solubility. He atoms diffuse and form clusters with vacancies, forming bubbles. It is believed that at high temperatures of 773–973 K and high helium ion energy, helium diffusion and bubble formation in FCC HEAs are slowed down compared to pure Ni due to the suppression of thermal vacancy formation and enhancement of defect recombination [8]. Helium bubbles can grow into voids, leading to surface erosion, when the surface swells and large bubbles (blisters) are formed and exfoliation of the surface layer of the target sample occurs (flaking) [9,10]. All these phenomena lead to radiation-induced creep, cracking, fatigue, and the deterioration of thermophysical properties of structural materials [1]. Therefore, it is important to study the stability of the solid solution and resistance to blistering of HEAs at high temperatures close to the melting point.

HEAs and similar concentrated solid solutions (CSS) with similar compositions are more resistant to helium swelling and embrittlement than pure nickel and steels. For example, in the CSS NiCoFeCr, smaller helium bubbles are formed compared to nickel when irradiated with helium ions at 500 °C, and the helium bubble size increases with fluence [11]. In Ni-based HEAs, helium bubbles grow in size with increasing temperature, while the number density of these bubbles decreases [12,13]. In the FCC HEAs NiCoFeCr, NiCoFeCrMn, when irradiated with helium at high temperatures of 500–700 °C, the number density of helium bubbles is higher and the bubble size is smaller than in pure nickel or austenitic steels [8,14]. With increasing helium ion fluence, the number density of helium bubbles in CoCrFeMnNi also increases, but it decreases with increasing temperature [14]. 

Helium bubbles and other radiation damage affect the microstrain and lattice stresses of alloys. The formation of helium bubbles and blisters makes a significant contribution to the lattice deformation of crystals and fatigue of metals [9]. When equiatomic alloys and HEAs are irradiated with Ni and He ions, the thermal conductivity decreases and the local temperature increases due to electron scattering by the lattice defects, and the recombination of defects, the creation of small defect clusters, and annealing of defects occur, which ultimately lead to stress relaxation [15,16]. It was shown that the elastic lattice strain in NiFeCoCr was larger than in nickel when irradiated with Ni ions, but at low irradiation energies the strain increased from 0 to 0.03%, and at high energies it decreased from 0.03 to 0.02% with increasing fluence [15]. When nanocrystalline Ni was irradiated, the coherent scattering region (CSR) size decreased with increasing dose, since stable vacancy clusters were formed and the dislocation loop density increased, but changes in microstrain were minimal, and the average strain at a fluence of 2.3 × 10^16^ cm^−2^ reached 1.5 × 10^−3^ [17]. When Inconel 625 alloy was irradiated with 5 MeV helium ions with increasing fluence, the CSR size also decreased, and the microstrain increased from 1.8 × 10^−3^ to 4.2 × 10^−3^, due to the accumulation of point defects, but at a fluence of 10^15^ cm^−2^, the CSR size increased, and the microstrain slightly decreased, which is associated with further annealing of defects [18]. When nickel-based alloy 617 was irradiated with 540 keV helium ions at 700 °C at low doses of 0.5–2 dpa, the vacancy defects such as helium bubbles created small strain, and the hardness increased slightly [19]. Thus, due to the annealing of defects and the reduction of thermal conductivity, stress relaxation and stress reduction can occur.

The HEAs are also resistant to forming large defect clusters compared with binary nickel alloy systems and have the self-healing effect. Dislocation loops and lines can significantly affect the hardening and embrittlement, but in CrMnFeCoNi and CrFeCoNi, the density of dislocation loops after irradiation by Ni ions was higher than that of binary and ternary nickel alloys, and most of these loops had a smaller size [5]. The reasons for the self-healing of HEAs include high atomic stress and lattice distortion caused by the large difference in atomic size, which lead to the amorphization followed by crystallization of the alloy [20,21], and the similarity of vacancy and interstitial migration energies, which improve the defect annihilation [11]. Thus, the structural features of the HEAs affect the radiation resistance.

In addition to bubble formation and strain, segregation of HEA elements can occur under helium ion irradiation. Lattice distortion and lattice stress affect the formation and migration energies of point defects and HEA atoms [8]. At the same time, helium bubble formation and blistering affect the motion of vacancies and strain fields [9]. Radiation-induced segregation occurs due to different diffusion coefficients, migration energies, concentrations of certain defects and their sinks, and changes in grain boundaries [5,22]. In Ni–Co–Fe–Cr–Mn system alloys, Mn concentrations often decrease and Ni/Co concentrations increase [5]. When annealed at high temperatures, NiCoFeCrMn exhibits the stability of the main FCC phase, but at intermediate temperatures (773–973 K) during long-term annealing, phases with a high concentration of Cr, NiMn, and FeCo are formed [23,24].

Studies of radiation resistance of HEAs often focus on studying the properties and structure, distribution of defects by depth of samples of only one HEA with an FCC lattice, when irradiated with various fluences and ions, including Ni self-ions, heavier particles, and helium. Studies of the effect of helium on the macro- and microstresses and surface morphology of both NiCoFeCrMn and NiCoFeCr HEAs simultaneously are rare. In the authors’ previous work [25], the radiation resistance of CoCrFeNi, CoCrFeMnNi HEAs, and pure Ni was compared under irradiation with 40 keV helium ions, with an increase in the fluence of helium ions to 2 × 10^17^ cm^−2^ at room temperature. 

This work aimed to study and compare the radiation resistance of CoCrFeNi and CoCrFeMnNi HEAs under irradiation with helium ions at 700 °C, as well as to identify the mechanisms of radiation-induced defects’ behavior, which is a continuation of the study of the effect of helium irradiation. Structural studies were mainly conducted using the X-ray diffraction method.

## 2. Materials and Methods

**Figure 1:**
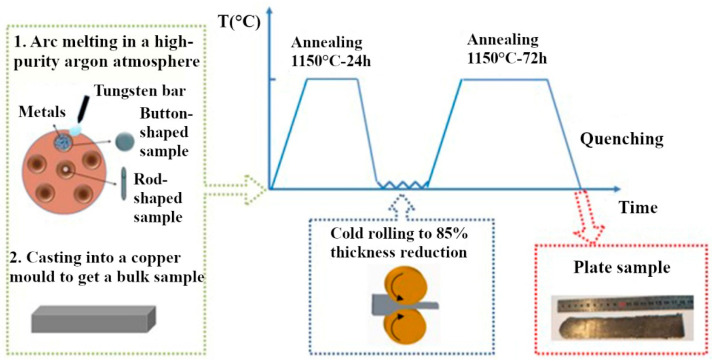
FCC high-entropy alloy sample preparation diagram [25].

High-entropy alloys CoCrFeNi and CoCrFeMnNi and a reference Ni sample were manufactured at the Beijing Institute of Technology (Beijing, China) using the same procedure as in our previous study [25]. Powders of metals with a purity of up to 99.97% underwent arc melting in the atmosphere of argon with high purity and were cast into a copper mold to obtain bulk ingots. To obtain a spheroidized and homogeneous grain structure of the samples, the ingots were annealed for 24 h at 1150 °C (Figure 1) after the crystallization of ingots. To reduce the thickness of ingots by 85%, they went through cold-rolling and, at the end, annealed at 1150 °C for 72 h to decrease the amount of texture and stresses caused by rolling. The fraction of the crystallization temperature (*T_r_*) of CoCrFeMnNi and CoCrFeNi HEA was 0.89*T_r_* and 0.81 *T_r_*, respectively. All obtained samples were in the form of rectangular parallelepipeds with dimensions of 5.0 × 5.0 × 1.5 mm.

The irradiation of samples was conducted at a DC-60 heavy ion accelerator located in the Astana branch of the Institute of Nuclear Physics (Astana, Kazakhstan). CoCrFeNi, CoCrFeMnNi, and Ni samples were irradiated by He^2+^ ions with an energy of 40 keV at a fluence of ions of 2 × 10^17^ cm^−2^ at a temperature of 700 °C using a target holder with heater.

Scanning electron microscopy with a 20 kV accelerating voltage on a ZEISS LEO 1455 VP microscope (Jena, Germany) was used to analyze the morphology and obtain the images of the surfaces of HEA and Ni samples. The diameter of pores was calculated from SEM images using ImageJ software (version 1.54d) [26] and plotted as histograms. The composition of unirradiated and irradiated samples was studied using the energy-dispersive spectroscopy method on the given microscope.

Phase and structure studies were carried out using X-ray diffraction (XRD) analysis. Rigaku Ultima IV X-ray diffractometer was used to acquire the X-ray diffraction patterns in parallel beam geometry, utilizing CuKα characteristic X-ray radiation with a wavelength λ = 0.154179 nm. To reduce the effect of alloy texture, the samples were constantly rotated at a rate of 30 rps during X-ray pattern acquisition. To focus on studying the irradiated region of the samples, a grazing incidence X-ray diffraction (GIXRD) mode at an angle of incidence α of the X-ray beam was used. 

Residual stresses can be characterized by the scale at which they exist within a material. Stresses that occur over long distances within a material are referred to as macrostresses. Stresses that exist only locally (either between grains or inside a grain) are called microstresses. The g-sin^2^ψ method [27] was applied to calculate the macrostresses. The method allows calculating macrostresses in the near surface layers by changing the incidence angle α and depth of scanning of the X-ray beam. The Williamson–Hall method [28] was used to estimate the microstresses and density of dislocations, and this method allows for separating the contribution of coherent scattering regions (CSRs) and microstrain on a diffraction peak broadening. The density of dislocations (ρ) was estimated by the formula ρ = 3/D^2^ [29], where D is the size of CSR.

## 3. Results and Discussion

### 3.1. Structure and Elemental Composition of As-Prepared CoCrFeNi and CoCrFeMnNi HEAs

The results of the study of the surface microstructure, composition, and structure of the initial CoCrFeNi and CoCrFeMnNi alloys are presented in our previous work [25]. The alloys are single-phase solid solutions of (Co, Cr, Fe, Ni) and (Co, Cr, Fe, Mn, Ni) with an FCC lattice, a coarse-grained structure (80–100 μm), and a uniform distribution of elements by depth. 

In the initial CoCrFeNi and CoCrFeMnNi alloys, tensile macro- (103 ± 10 and 44 ± 5 MPa) and microstresses (1.05 ± 0.15 and 0.88 ± 0.15 GPa, respectively) were revealed [25], the appearance of which is associated with the mechanical processing of materials at the manufacturing stage.

### 3.2. Structure and Elemental Composition of CoCrFeNi and CoCrFeMnNi HEAs Irradiated by Helium Ions at 700 °C

According to calculations using the Stopping and Range of Ions in Matter (SRIM-2013) program [30] of radiation damage in CoCrFeNi and CoCrFeMnNi HEAs, the projective range of helium ions in the samples is 146 nm, and the maximum energy loss in the region up to 100 nm is 0.22 keV/nm. The maximum concentration of implanted helium and the damaging dose in nickel and HEAs are approximately equal and amount to 16 at.% and 23 dpa for an irradiation fluence of 2 × 10^17^ cm^−2^, respectively [25]. However, the SRIM calculations are provided for room temperature and do not take into account the heating effects.

The results of studying the elemental composition of CoCrFeNi and CoCrFeMnNi HEAs irradiated by He ions are shown in Table 1.

As can be seen from Table 1, high-temperature irradiation with helium ions does not lead to a significant change in the elemental composition of the CoCrFeNi alloy. At the same time, irradiation of the CoCrFeMnNi alloy leads to an increase in the concentration of Co, Cr, and Fe, a small increase in the concentration of nickel, and a decrease in the concentration of manganese by 4 at.%.

Changes in the composition of the HEAs are believed to be mainly associated with the formation of radiation defects and their interaction with thermal vacancies. Since at the final stage of formation HEA samples were annealed at 1150 °C for 72 h, vacuum annealing at 700 °C will not significantly change their elemental composition (or these changes will be within the error limits). The above is confirmed by the results of the article [31], which shows that long-term (96 h) vacuum annealing at 1100 °C does not lead to a change in the elemental composition of the CoCrFeMnNi HEA. Therefore, the authors believe that changes in the composition of the HEAs are mainly associated with the formation of radiation defects during irradiation with helium ions. In the previous work [25], the authors showed changes in the composition of CoCrFeNi and CoCrFeMnNi HEAs after irradiation with helium ions with an energy of 40 keV and fluences of up to 2 × 10^17^ cm^−2^ at room temperature. The magnitudes of these changes are comparable with the data obtained at high-temperature irradiation with helium ions.

A slight decrease in the Fe concentration in the CoCrFeNi HEA (0.4 at.%) is probably due to the migration of Fe atoms from the surface layer deeper into the sample due to the exchange with vacancies from a greater depth, as shown in [32] when irradiating NiCoFeCr with Ni ions at a temperature of 500–580 °C. The diffusion of atoms can also be affected by the lattice distortion and the configuration of d-shell electrons [11]. In addition, dislocation loops, helium bubbles, and other defect clusters in the HEA can affect the diffusion rate of elements [33,34]. 

The decrease in the Mn concentration in the CoCrFeMnNi HEA (4 at.%) is probably associated with radiation-induced segregation processes along the grain boundaries, as shown in [5], where Mn concentrations decrease and Ni/Co concentrations increase in Ni–Co–Fe–Cr–Mn alloys upon irradiation with nickel ions. Moreover, [35] showed that the diffusion coefficients by the vacancy mechanism in the CoCrFeNiMn HEA irradiated with Ni at a temperature of 500 °C decrease from Mn to Cr, Fe, and Ni, which is the least mobile of them with Co, which causes a sharp decrease in Mn near the grain boundaries.

Thus, high-temperature irradiation with helium ions does not lead to a significant change in the concentration of elements in the irradiated samples.

**Figure 2:**
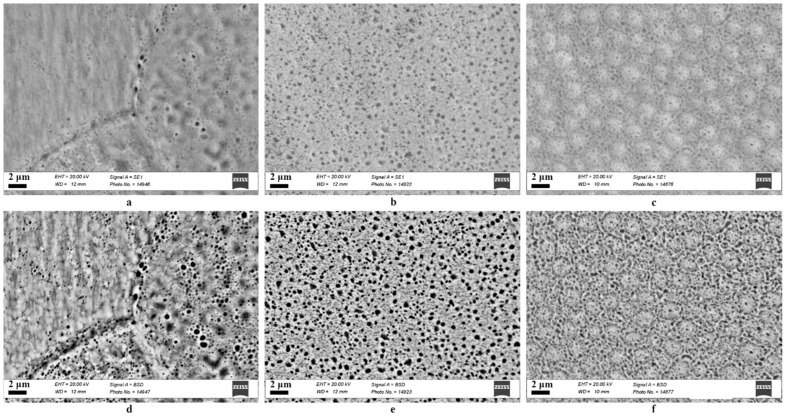
SEM: (**a**–**c**) secondary electron images and (**d**–**f**) backscattered electron images of the surface of: (**a**,**d**) Ni, (**b**,**e**) CoCrFeNi, (**c**,**f**) CoCrFeMnNi, irradiated with helium ions.

**Figure 3:**
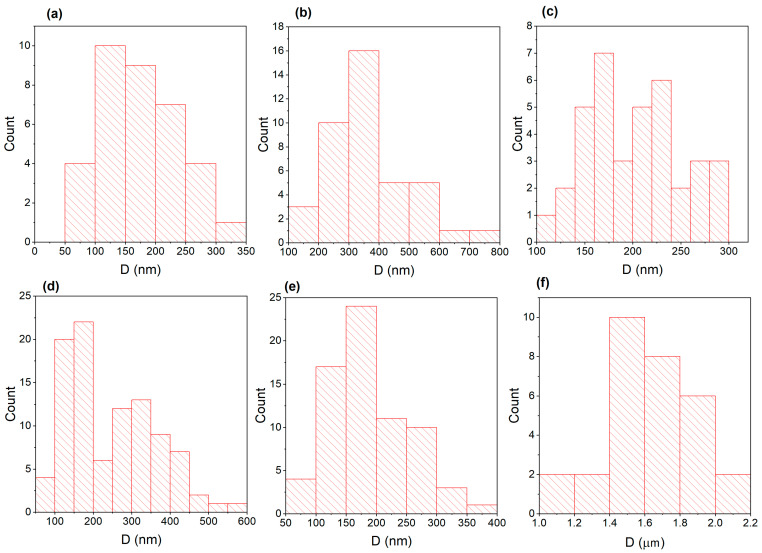
Histograms of the pore diameter obtained from the SEM images for: (**a**) Figure 2d, left grain of Ni, (**b**) Figure 2d, right grain of Ni, (**c**) Figure 2d, lower located grain of Ni, (**d**) Figure 2e, CoCrFeNi and (**e**) Figure 2f, CoCrFeMnNi samples irradiated with helium ions; (**f**) Figure 2f, diameter of the swellings of irradiated CoCrFeMnNi.

The results of the study of the surface of the samples after high-temperature irradiation with helium ions are shown in Figure 2. As can be seen from the figures, blisters in the form of closed (light spots) and open (dark spots) pores/channels are present on the surface of all samples. It is known [10] that irradiation with helium ions at a temperature equal to half the melting point of the alloy can cause helium bubbles to form channels and the surface to become porous. As can be seen from Figure 2 and Figure 3, a large portion of pores in the CoCrFeNi alloy have a diameter of 100–400 nm (Figure 3d), while in CoCrFeMnNi most of the pores have a diameter of 100–300 nm and reach up to 400 nm (Figure 3e). The obtained data indicate a decrease in the size of blisters in the CoCrFeMnNi alloy compared to CoCrFeNi by 1.3 times the average diameter. At the same time, pores with a diameter of 100 nm and less are also present in these alloys. It is worth noting the formation of swellings with a diameter of about 1.4–2 μm (Figure 2f and Figure 3f) on the surface of the CoCrFeMnNi HEA. However, the analysis of images obtained in backscattered electron mode does not indicate the formation of gas cavities underneath them. Figure 2a,d shows how the orientation of the grains of nickel affects the formation of blisters (channels) in them. In the left grain, closed blisters with a diameter of 50–250 nm (white spots) are visible. There are also rare open blisters (channels) with a diameter reaching 350 nm. In the right grain, larger blisters are observed, where a significant portion of them has a diameter of 500–700nm. In the lower grain, blisters with a diameter of 150–270 nm are more frequent.

**Figure 4:**
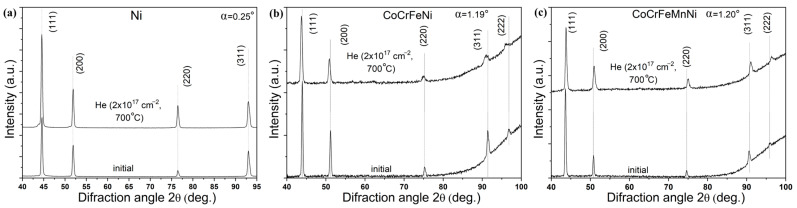
GIXRD patterns of initial and irradiated with helium ions samples of: (**а**) Ni, (**b**) CoCrFeNi and (**c**) CoCrFeMnNi HEAs, obtained at the angle of incidence α of X-ray radiation.

The results of the study of the phase composition of the HEAs after high-temperature irradiation with helium ions are presented in Figure 4. X-ray diffraction patterns were obtained at small angles of incidence α = 0.25°, 1.19° and 1.20° of X-ray radiation for Ni, CoCrFeNi, and CoCrFeMnNi, respectively. Angles α= 0.25°, 1.19° and 1.20° correspond to the penetration depth of X-ray radiation equal to 300 nm. The X-ray patterns contain diffraction peaks corresponding to reflections from the planes of the fcc lattices. For all samples, the preferred orientation is (111).

Analysis of GIXRD patterns of samples after high-temperature irradiation with helium ions (Figure 4) did not reveal the appearance of diffraction peaks corresponding to new phases or the disappearance of existing ones, i.e., no decomposition of solid solutions (Co, Cr, Fe, Ni) and (Co, Cr, Fe, Mn, Ni) occurred. This indicates high radiation resistance of the phase composition of the HEAs to high-temperature irradiation with helium ions with a fluence of 2 × 10^17^ cm^−2^. 

High-temperature irradiation does not change the angular position of the diffraction peaks of Ni within the error limits (Figure 4a) but leads to a shift of the diffraction peaks of solid solutions (Co, Cr, Fe, Ni) to the region of smaller angles 2θ (Figure 4b), and solid solutions (Co, Cr, Fe, Mn, Ni) to the region of larger angles 2θ (Figure 4c). Calculations showed that high-temperature irradiation leads to an increase in the lattice parameter of the solid solution (Co, Cr, Fe, Ni) by 0.40% and a decrease in the lattice parameter of (Co, Cr, Fe, Mn, Ni) by 0.53%. The accuracy of determining the lattice parameter is 0.02–0.03%. The lack of peak shift in Ni is probably because of the annihilation of radiation defects and their clustering into helium bubbles competing with each other, as in helium-irradiated nanocrystalline Ni [17].

Another study showed that the lattice deformation in NiFeCoCr is larger than that in nickel under Ni ion irradiation [15]. Based on the literature, the increase in the lattice parameter of CoCrFeNi could be caused by the accumulation of point defects and their small clusters, which is limited by the annealing of defects [18,36], but it would still remain a microscopic type of swelling [16]. At the same time, high-temperature irradiation improves the recombination of defects and reduces the tensile lattice deformations, so in CoCrFeMnNi HEA, the lattice shrinking could be due to its distorted lattice relaxation [15,16]. The HEAs’ response differed from that of nickel under irradiation, probably due to their distorted lattice.

For a more detailed understanding of the mechanisms of formation and evolution of radiation defects under high-temperature irradiation with helium, studies of macro- and microstresses and dislocation density were conducted in the region before the peak of implanted helium ions (region I—up to 100 nm) and in the region of the peak of implanted helium ions (region II—from 100 to 300 nm).

**Figure 5:**
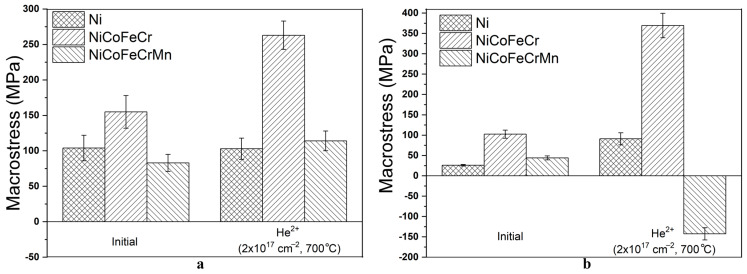
Macrostresses in initial and irradiated with helium ions samples of Ni, CoCrFeNi and CoCrFeMnNi HEAs at X-ray penetration depths of: (**a**) 100 nm and (**b**) 300 nm.

Studies of macrostresses in samples were conducted using the g-sin^2^ψ method to evaluate the change in macrostresses after high-temperature irradiation. The results of determining the macrostresses in the initial and irradiated samples of Ni, CoCrFeNi, and CoCrFeMnNi HEAs at α = 0.084° and 0.25°, 0.39° and 1.19°, and 0.39° and 1.20°, calculated for the plane (111), are shown in Figure 5. The angles of incidence of X-ray radiation α = 0.084° and 0.39° correspond to a penetration depth of 100 nm.

As can be seen from Figure 5a, in region I, high-temperature irradiation of CoCrFeNi and CoCrFeMnNi HEAs with helium ions leads to an increase in tensile macrostresses in them by 1.7 and 1.4 times, respectively. At the same time, the level of tensile macrostresses in Ni remains constant within the error limits (103 MPa). The formation of tensile macrostresses in region I is probably associated with the high irradiation temperature and the formation of many thermal vacancies, which could lead to the annihilation of radiation defects and the formation of a porous surface structure. High-temperature irradiation with helium ions leads to the formation of a porous structure of the nickel surface, which could contribute to the effective removal of radiation defects and their weak accumulation in the area of irradiation with helium ions. This would not lead to an increase in the macrostress level in irradiated Ni and would maintain it close to the value of the original sample.

In region II, tensile macrostresses in Ni and CoCrFeNi HEAs increase by 3.6 times (Figure 5b). At the same time, compressive macrostresses of −143 MPa are formed in CoCrFeMnNi HEAs. The obtained data seem to indicate the formation of helium-vacancy clusters and helium bubbles in CoCrFeMnNi HEAs in region II, probably leading to the formation of compressive macrostresses. Meanwhile, for CoCrFeNi HEAs, the macrostresses remain tensile. This could point to a high intensity of radiation defect diffusion processes in these alloys. Based on similar research [5], it can be assumed that in CoCrFeMnNi HEA there could be a partial accumulation of radiation defects and a slowdown in helium diffusion due to a large lattice distortion. The formation of helium bubbles in CoCrFeMnNi could be confirmed by the formation of swellings on the surface of these alloys.

**Figure 6:**
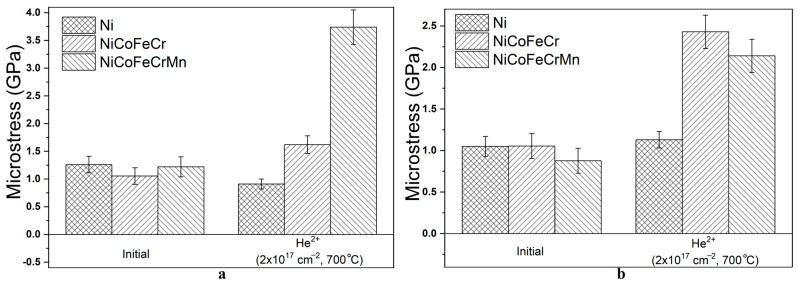
Microstresses in initial and irradiated with helium ions samples of Ni, CoCrFeNi, and CoCrFeMnNi HEAs at X-ray penetration depths of: (**a**) 100 nm and (**b**) 300 nm.

The analysis of microstresses of irradiated samples (Figure 6) showed that high-temperature irradiation with helium ions leads to a change in the values of tensile microstresses compared to the initial microstresses. In region I, microstresses in CoCrFeNi and CoCrFeMnNi increase by 1.5 and 3.1 times, respectively. In region II, microstresses in CoCrFeNi and CoCrFeMnNi increase by 2.4 times. At the same time, the level of tensile microstresses in region II is lower than in region I. For Ni samples, the level of tensile microstresses in region I decreases by 28%, and in region II it increases by 7.6%.

The obtained data on microstresses in region I are similar to the data on irradiation with helium ions at room temperature [25], where the level of microstresses in the HEAs was about 2 GPa. However, the level of microstresses in region II under high-temperature irradiation is 2–2.5 times lower than under room-temperature irradiation with helium ions [25]. This is probably due to the formation and annihilation of radiation defects at high temperature (700 °C). At the same time, the distortion of the HEA lattice could also affect the formation of microstrain [15] and microstress. Thus, for the Ni sample, we see a decrease in microstresses by 2–2.5 times under high-temperature irradiation compared to irradiation at room temperature [25].

Another reason for the changes in microstresses could lie in the influence of helium. According to the work [8], at high temperatures of 773–973 K and high helium ion energy, helium diffusion and bubble formation in CoCrFeNi, CoCrFeMnNi HEAs slow down due to the suppression of the formation of thermal vacancies and the enhancement of defect recombination, but the bubble size increases, although it remains smaller than in Ni.

**Figure 7:**
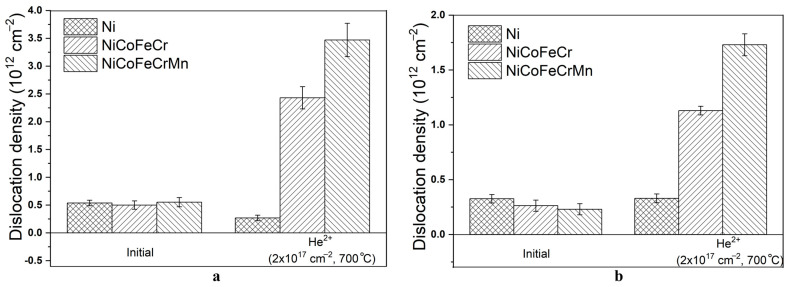
Density of dislocations in initial and irradiated with helium ions samples of Ni, CoCrFeNi, and CoCrFeMnNi HEAs at X-ray penetration depths of: (**a**) 100 nm and (**b**) 300 nm.

The results of determining the dislocation density in samples for regions I and II are presented in Figure 7. The size of the CSR in the samples was 18–65 nm.

As can be seen from Figure 7, high-temperature irradiation with helium ions leads to an increase in the dislocation density by 4.9 and 6.3 times in region I for the CoCrFeNi and CoCrFeMnNi HEAs, respectively. In region II, an increase in the dislocation density by 4.3 and 7.5 times was also obtained for the CoCrFeNi and CoCrFeMnNi HEAs, respectively. In the case of Ni samples, the dislocation density in region I decreases by 50%, and in region II it increases by 12%.

The values of the dislocation density in region I for the HEA are close to the data on irradiation with helium ions at room temperature [25], where the dislocation density value is (2–2.5) ×10^12^ cm^−2^. However, the dislocation density values in region II under high-temperature irradiation are 8–10 times lower than under room-temperature irradiation with helium ions [25]. This, as in the case of microstresses, could be associated with the formation of a porous structure of the HEA under high-temperature irradiation. At the same time, the distortion of the HEA lattice also affects the value of the dislocation density. Thus, for the Ni sample, we see a decrease in the dislocation density by 3–10 times under high-temperature irradiation compared to irradiation at room temperature [25]. A significant drop in the dislocation density in the Ni samples could be probably explained by the high irradiation temperature. It was shown [14] that in Ni, the diffusion of point defects in one dimension over a long distance was dominant, which could lead to a decrease in the dislocation density in Ni. 

In CrFeCoNi, the formation of dislocation loops is also weakened compared to nickel, probably due to the enhancement of vacancy and interstitial recombination [5]. In CrMnFeCoNi, when irradiated with nickel ions at 500 °C to 38 dpa, the dislocation loop density was higher than in CrFeCoNi, and most loops were smaller [33]. Since the radiation damage dose from helium in CoCrFeNi/CrMnFeCoNi is comparable and equal to 23 dpa, the dislocation density of CrMnFeCoNi can be higher than in the quaternary HEA, but this also depends on their depth distribution and the calculation method [5]. In addition, the increase in the dislocation loop density is probably due to the higher density of helium bubbles in CoCrFeMnNi than in Ni and CoCrFeNi, as reported by other authors, due to the combination of vacancies with helium and a decrease in interstitial annihilation [5,14]. At the same time, the dislocation densities in Ni, CoCrFeNi, and CoCrFeMnNi are still lower than those under helium irradiation at room temperature. This is probably due to the enhancement of defect recombination, a decrease in the number of helium bubbles, and the annihilation of defects in sinks [5,12]. Thus, high temperature and bubble growth reduce the dislocation density in Ni and CoCrFeNi, while the growth in CoCrFeMnNi is insignificant.

The elemental and phase composition of the considered CoCrFeNi and CoCrFeMnNi HEAs were shown to be resistant to irradiation with helium ions (40 keV, 2 × 10^17^ cm^−2^) at a temperature of 700 °C; no formation of new phases was detected. The main changes obtained as a result of irradiation of the samples are associated with changes in the dislocation density, as well as the formation and redistribution of stresses in the alloys. 

The data on the change in macro- and microstresses and dislocation density indicate high radiation resistance of HEAs under high-temperature irradiation with helium ions compared to nickel. Analysis of these data also shows a higher resistance of CoCrFeMnNi HEAs to radiation-induced changes in the microstructure and stresses compared to CoCrFeNi.

## 4. Conclusions

It was found that the irradiation of CoCrFeNi and CoCrFeMnNi HEAs by He^2+^ ions of 40 keV energy at a fluence of 2 × 10^17^ cm^−2^ and a temperature of 700 °C does not lead to changes in the elemental and phase composition. A slight decrease in the Fe concentration in CoCrFeNi HEA (0.4 at.%) and Mn in CoCrFeMnNi HEA (4 at.%) is observed. An increase in the lattice parameter of the (Co, Cr, Fe, Ni) solid solution by 0.40% and a decrease in the lattice parameter of (Co, Cr, Fe, Mn, Ni) by 0.53% are established. 

It was revealed that in the region before the peak of implanted helium, high-temperature irradiation of CoCrFeNi and CoCrFeMnNi with helium ions leads to an increase in tensile macrostresses in them by 1.7 and 1.4 times, an increase in tensile microstresses by 1.5 and 3.1 times, and an increase in the dislocation density by 4.9 and 6.3 times, respectively. 

In the region of the peak of implanted helium, tensile macrostresses in CoCrFeNi increase by 3.6 times, and in CoCrFeMnNi compressive macrostresses are formed, amounting to −143 MPa. Microstresses in the HEAs increase by 2.4 times, and the dislocation density value increases by 4.3 and 7.5 times for the HEA of CoCrFeNi and CoCrFeMnNi, respectively. 

It was found that high-temperature irradiation of CoCrFeNi and CoCrFeMnNi HEAs with He^2+^ ions leads to the formation of a porous surface structure by creating a large number of microchannels (open blisters). The diameter of the blisters in CoCrFeMnNi is 1.3 times smaller than in CoCrFeNi.

It was established that CoCrFeMnNi HEAs were characterized by greater radiation resistance compared to the CoCrFeNi alloy.

## Figures and Tables

**Table 1:** Elemental composition of CoCrFeNi and CoCrFeMnNi, initial and irradiated with helium ions.

Sample	Concentration of Elements, at.%				
	Co	Cr	Fe	Mn	Ni
CoCrFeNi (initial)	24.7 ± 0.1	25.7 ± 0.1	25.3 ± 0.1	-	24.3 ± 0.2
CoCrFeNi (Не^2+^)	24.8 ± 0.2	25.9 ± 0.1	24.9 ± 0.1	-	24.4 ± 0.2
CoCrFeMnNi (initial)	19.5 ± 0.2	20.3 ± 0.1	19.8 ± 0.2	20.6 ± 0.2	19.8 ± 0.2
CoCrFeMnNi (Не^2+^)	20.8 ± 0.2	21.3 ± 0.1	21.1 ± 0.1	16.6 ± 0.1	20.2 ± 0.2

## Data Availability

The authors declare that the data supporting this study are available from the corresponding author upon request.
